# The chest CT features of coronavirus disease 2019 (COVID-19) in China: a meta-analysis of 19 retrospective studies

**DOI:** 10.1186/s12985-020-01432-9

**Published:** 2020-10-21

**Authors:** Haitao Yang, Yuzhu Lan, Xiujuan Yao, Sheng Lin, Baosong Xie

**Affiliations:** Department of Pulmonary and Critical Care Medicine, Shengli Clinical Medical College of Fujian Medical University, Fujian Provincial Hospital, Dongjie Road No. 134, Fuzhou, 350001 Fujian China

**Keywords:** COVID-19, Coronavirus, Chest CT findings, Meta-analysis

## Abstract

**Objective:**

Aimed to summarize the characteristics of chest CT imaging in Chinese hospitalized patients with Coronavirus Disease 2019 (COVID-19) to provide reliable evidence for further guiding clinical routine.

**Methods:**

PubMed, Embase and Web of Science databases were searched to identify relevant articles involving the features of chest CT imaging in Chinese patients with COVID-19. All data were analyzed utilizing R i386 4.0.0 software. Random-effects models were employed to calculate pooled mean differences.

**Results:**

19 retrospective studies (1332 cases) were included. The results demonstrated that the combined proportion of ground-glass opacities (GGO) was 0.79 (95% CI 0.68, 0.89), consolidation was 0.34 (95% CI 0.23, 0.47); mixed GGO and consolidation was 0.46 (95% CI 0.37; 0.56); air bronchogram sign was 0.41 (95% CI 0.26; 0.55); crazy paving pattern was 0.32 (95% CI 0.17, 0.47); interlobular septal thickening was 0.55 (95% CI 0.42, 0.67); reticulation was 0.30 (95% CI 0.12, 0.48); bronchial wall thickening was 0.24 (95% CI 0.11, 0.40); vascular enlargement was 0.74 (95% CI 0.64, 0.86); subpleural linear opacity was 0.28 (95% CI 0.12, 0.48); intrathoracic lymph node enlargement was 0.03 (95% CI 0.00, 0.07); pleural effusions was 0.03 (95% CI 0.02, 0.06). The distribution in lung: the combined proportion of central was 0.05 (95% CI 0.01, 0.11); peripheral was 0.74 (95% CI 0.62, 0.84); peripheral involving central was 0.38 (95% CI 0.19, 0.75); diffuse was 0.19 (95% CI 0.06, 0.32); unifocal involvement was 0.09 (95% CI 0.05, 0.14); multifocal involvement was 0.57 (95% CI 0.48, 0.68); unilateral was 0.16 (95% CI 0.10, 0.23); bilateral was 0.83 (95% CI 0.78, 0.89); The combined proportion of lobes involved (> 2) was 0.70 (95% CI 0.61, 0.78); lobes involved (≦ 2) was 0.35 (95% CI 0.26, 0.44).

**Conclusion:**

GGO, vascular enlargement, interlobular septal thickening more frequently occurred in patients with COVID-19, which distribution features were peripheral, bilateral, involved lobes > 2. Therefore, based on chest CT features of COVID-19 mentioned, it might be a promising means for identifying COVID-19.

## Introduction

The Coronavirus disease 2019 (COVID-19) is caused by SARS-CoV-2, a new coronavirus of the Sarbe virus subgenus, a member of the orthocoronavirus subfamily [1]. The outbreak of the COVID-19 has resulted in a global pandemic. Up to August 16, 2020, a total of 21,294,865 confirmed cases have been reported in the world, with another 761,779 confirmed deaths [2]. Considering COVID-19 has caused a big threat to global health, WHO announced the event constituted a Public Health Emergency of International Concern (PHEIC), on December 30, 2019. Interrupting the spread of the pandemic has become an urgent problem. In the prevention and treatment of SARS-CoV-2, the "four early" principles (early detection, early diagnosis, early isolation and early treatment) are particularly important. Patients infected with SARS-CoV-2 may have fever, cough, dyspnea, and muscle pain, which are nonspecific [3–6]. However, the varieties of clinical manifestations, laboratory tests and imaging tests limit clinical diagnosis and treatment. As we all know, real-time reverse transcription-polymerase chain reaction (rRT-PCR) is the reference standard [6, 7]. However, nucleic acid testing is highly laboratory demanding, the long time for results and the false-negative results are harmful for the control of infectious diseases. Moreover, subclinical cases increase the difficulty of diagnosis. Some studies have shown that COVID-19 may have no clinical manifestations, but can find abnormal signals in chest CT [8–10]. Imaging can be used to guide diagnosis early in the course of the disease or in asymptomatic patients. Chest CT played an important role in timely detecting lung abnormalities, allowing for early treatment. Previous studies focused on the features of CT imaging of COVID-19, whereas the results varieties of different studies. Therefore, it is urgent to conduct this meta-analysis to comprehensively summarize the characteristics of CT imaging of patients with COVID-19 to further guide clinical and scientific research through evidence-based medicine.

## Material and methods

### Search strategy

Relevant articles were thoroughly searched from PubMed, Embase and Web of Science databases using the following words: "2019-nCoV", "Coronavirus", "COVID-19", "SARS-CoV-2", "Chest computer tomography (CT) manifestations", "Imaging findings", "China", "Chinese". Articles were dated up to 13 May 2020. The language was restricted to English. The identified articles with the references were also searched for extending the search. All recruited articles were performed by two researchers.

### Study selection

All articles meeting the following criteria were identified in this study: (1) all patients with COVID-19 were proved by RT-PCR; (2) all articles investigated the features of chest CT imaging with sufficient data; (3) all patients were Chinese; (4) all articles were published in English. Reviews, letters, case reports, ongoing studies and studies with insufficient data were excluded.

### Data extraction

Two researchers extracted the data from eligible articles independently. The following data included clinical characteristics (author, published year, sample size, gender, age-range, fever, cough, myalgia or fatigue, sore throat, dyspnea, diarrhea, nausea, and vomiting, study type, a period of study, in-patients, asymptomatic, CT negative group, time of symptom onset to CT) and the features of chest CT [ground-glass opacities (GGO), consolidation, mixed GGO and consolidation, air bronchogram sign, crazy paving pattern, interlobular septal thickening, reticulation, bronchial wall thickening, vascular enlargement, subpleural linear opacity, intrathoracic lymph node enlargement, pleural effusions, central, peripheral, peripheral involving central, unilateral, bilateral, diffuse, unifocal involvement, multifocal involvement, number of lobes involved (> 2), number of lobes involved (≤ 2)]. The third researcher decided once the extracted data existed discrepancy.

### Statistical analysis

All data were analyzed utilizing R software version i386 4.0.0. Eligible data were first transformed by one of the methods [raw, i.e. untransformed, proportions (PRAW), log transformation (PLN), logit transformation (PLOGIT), arcsine transformation (PAS), Freeman–Tukey double arcsine transformation (PFT)] to make them conform to normal distribution. Random-effects models were employed to calculate pooled mean differences due to the existed incorporate heterogeneity.

### Quality assessment

The quality evaluation of the article was conducted by two investigators independently according to the Newcastle–Ottawa Scale (NOS). When the results were inconsistent, discussion or decision would be made by the third investigator. 8 items from the three aspects of crowd selection, comparability and exposure, which was evaluated by star rating. The full score is 9 stars, ≥ 6 stars were regarded as high quality article, otherwise, low quality article.

### Publication bias

A minimum of 10 studies was needed to assess the potential publication bias, therefore we conducted the Egger test to assess this publication bias.

## Results

### Literature search and clinical characteristics

**Fig. 1:**
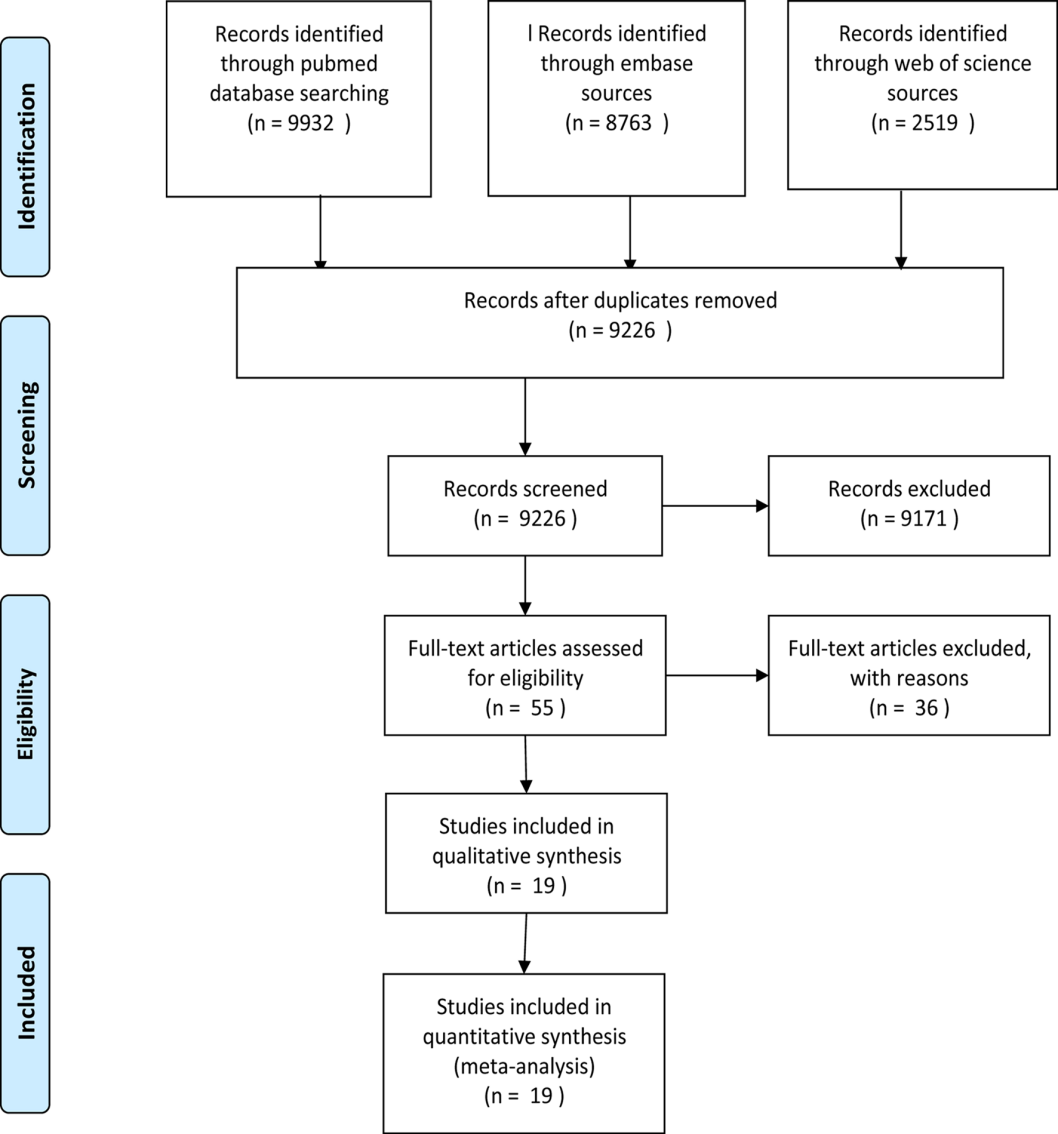
The Flow chart for study selection

Firstly, the above terms were used to comprehensively search from 3 databases and included 21,214 articles. Secondly, 11,988 articles were excluded by checking duplication. Furthermore, 9171 articles were excluded by reading abstract and title; 36 articles were excluded by reading full text (13 articles were case reports; 3 articles included only children or pregnant women; 3 articles were not Chinese). Eventually, 19 articles (1332 cases) [8, 11–28] were recruited to perform this meta-analysis to describe the chest CT feature of COVID-19. The process of searching was shown in Fig. 1, and the clinical features of the included studies was described in Tables 1 and 2. Quality evaluation of the included studies were described in Table 2. The exact search string and settings for each database (Additional file 1) and the 36 references of exclusion (Additional file 2) were described on the additional files.

**Table 1:** The characteristics of included studies

References	Sample size	Gender		Age range	Symptoms								
		Male	Female		Fever	Cough	Myalgia or fatigue	Sore throat	Dyspnea	Diarrhea	Nausea and vomiting	Sputum	Headache
Zhao et al. [11]	101	56	45	44.4 (17–75)	79	63	17	12	1	3	2	NA	NA
Xu et al. [12]	90	39	51	50 (18–86)	70	57	25	23	NA	5	2	NA	NA
Han et al. [13]	108	38	70	45 (21–90)	94	65	56	14	NA	15	NA	NA	NA
Li and Xia [14]	53	28	23	58 (26–83)	46	1	3	NA	NA	NA	NA	NA	NA
Xiong et al. [15]	42	25	17	49.5 (26–75)	36	27	14	NA	8	10	NA	NA	NA
Cheng et al. [16]	11	8	3	50.36	8	7	3	1	1	NA	NA	3	NA
Xu et al. [17]	50	29	21	13.9 (3–85)	43	20	16	4	4	1	1	NA	5
Yuan et al. [18]	27	12	15	60 (47–69)	21	16	3	NA	11	NA	NA	NA	NA
Chung et al. [19]	21	13	8	51 (29–77)	14	9	6	NA	NA	NA	1	NA	3
Zhang et al. [20]	17	8	9	48.6 (23–74)	12	9	13	1	1	NA	NA	7	4
Pan et al. [21]	63	33	30	44.9	NA	NA	NA	NA	NA	NA	NA	NA	NA
Shi et al. [8]	81	42	39	19.5 (25–81)	59	48	7	NA	34	3	4	15	5
Li et al. [22, 29]	90	44	39	45.5	72	65	15	6	9	7	NA	15	9
Bernheim et al. [23]	121	61	60	45.3 (18–80)	74	58	NA	NA	NA	NA	NA	20	NA
Wu et al. [24]	80	38	42	44	61	58	13	9	7	7	NA	11	8
Guan et al. [25]	53	25	28	42 (1–86)	NA	NA	NA	NA	NA	NA	NA	NA	NA
Bai et al. [26]	219	119	100	44.8 ± 14.5 (4–76)	142	NA	NA	NA	NA	NA	NA	NA	NA
Song et al. [28]	51	25	26	49 ± 16	49	24	16	3	7	5	3	NA	8
Miao et al. [27]	54	28	26	45.1 ± 13.4	NA	NA	NA	NA	NA	NA	NA	NA	NA

*NA* not available

**Table 2.** The characteristics of included studies and NOS score

Author	Study type	Province	Period of study	In-patients	Asymptomatic	CT negative	Group	Time of symptom onset to CT	NOS score
Zhao et al.	Retrospective	Hunan	NA	Hospitalised	2	8	Nonemergency Group (mild and common types), Emergency Group (severe and fatal types)	Range 0–7 days; median: 1 day	6
Xu et al.	Retrospective	Guangdong	2020/1/23–2020/2/4	Hospitalised	6	0	NA	NA	5
Han et al.	Retrospective	Hubei	2020/1/4–2020/2/3	Hospitalised	0	0	Mild	Range 1–3 days; median: 1 day	4
Yan Li	Retrospective	Hubei	2020/1/23–2020/1/29	Hospitalised	1	0	NA	NA	7
Xiong et al.	Retrospective	Hubei	2020/1/11–2020/2/5	Hospitalised	0	0	NA	Range 1–11 days; mean: 4.5 days	6
Cheng et al.	Retrospective	Shanghai	2020/1/19–2020/2/6	Hospitalised	0	0	NA	NA	7
Xu et al.	Retrospective	Beijing	2020/1–2020/2	Hospitalised	0	9	Mild, Moderate, severe/critically severe	NA	7
Yuan et al.	Retrospective	Hubei	2020/1/1–2020/1/25	Hospitalised	0	0	Survival group, Mortality group	Range 5–11 days; median: 8 days	8
Chung et al.	Retrospective	Guangdong, Jiangxi, Shangdong	2020/1/18–2020/1/27	Hospitalised	2	3	NA	NA	6
Zhang et al.	Retrospective	Sichuan	NA	Hospitalised	0	0	NA	Range 6 h-11 days; median:4.04 days	4
Pan et al.	Retrospective	Hubei	2019/12/30–2020/1/31	Hospitalised	NA	0	NA	NA	5
Shi et al.	Retrospective	Hubei	2019/12/20–2020/1/23	Hospitalised	0	0	NA	Group 1 (subclinical patients; scans done before symptom onset), group 2 (scans done ≤ 1 week after symptom onset), group 3 (> 1–2 weeks), group 4 (> 2–3 weeks)	8
Li et al.	Retrospective	Chongqing	2020/1–2020/2	Hospitalised	0	8	Ordinary group, severe/critical group	NA	6
Bernheim et al.	Retrospective	Jiangxi, Guangdong, Guangxi, Sichuan	2020/1/18–2020/2/2	Hospitalised	0	24	NA	Early phase: ≤ 2 days; intermediate pahse: 3–5 days; late phase:6–12 days	4
Wu et al.	Retrospective	Hubei	2020/1–2020/2	Hospitalised	0	14	NA	7 ± 4 days	4
Guan et al.	Retrospective	Beijing	2020/1/12–2020/2/28	Hospitalised	NA	6	NA	NA	6
Bai et al.	Retrospective	Hunan	2020/1/6–2020/2/20	Hospitalised	NA	37	NA	NA	6
Song et al.	Retrospective	Shanghai	2020/1/20–2020/1/27	Hospitalised	0	3	NA	Interval from disease onset to CT ≤ 4 Days; interval from disease onset to CT > 4 days	7
Miao et al.	Retrospective	Shanghai, Jiangxi	2020/1/12–2020/2/13	Hospitalised	0	28	NA	Less than 14 days	7

NA: not available

### The features of chest CT in COVID-19

#### GGO, consolidation, mixed GGO, and consolidation, air bronchogram sign

**Fig. 2:**
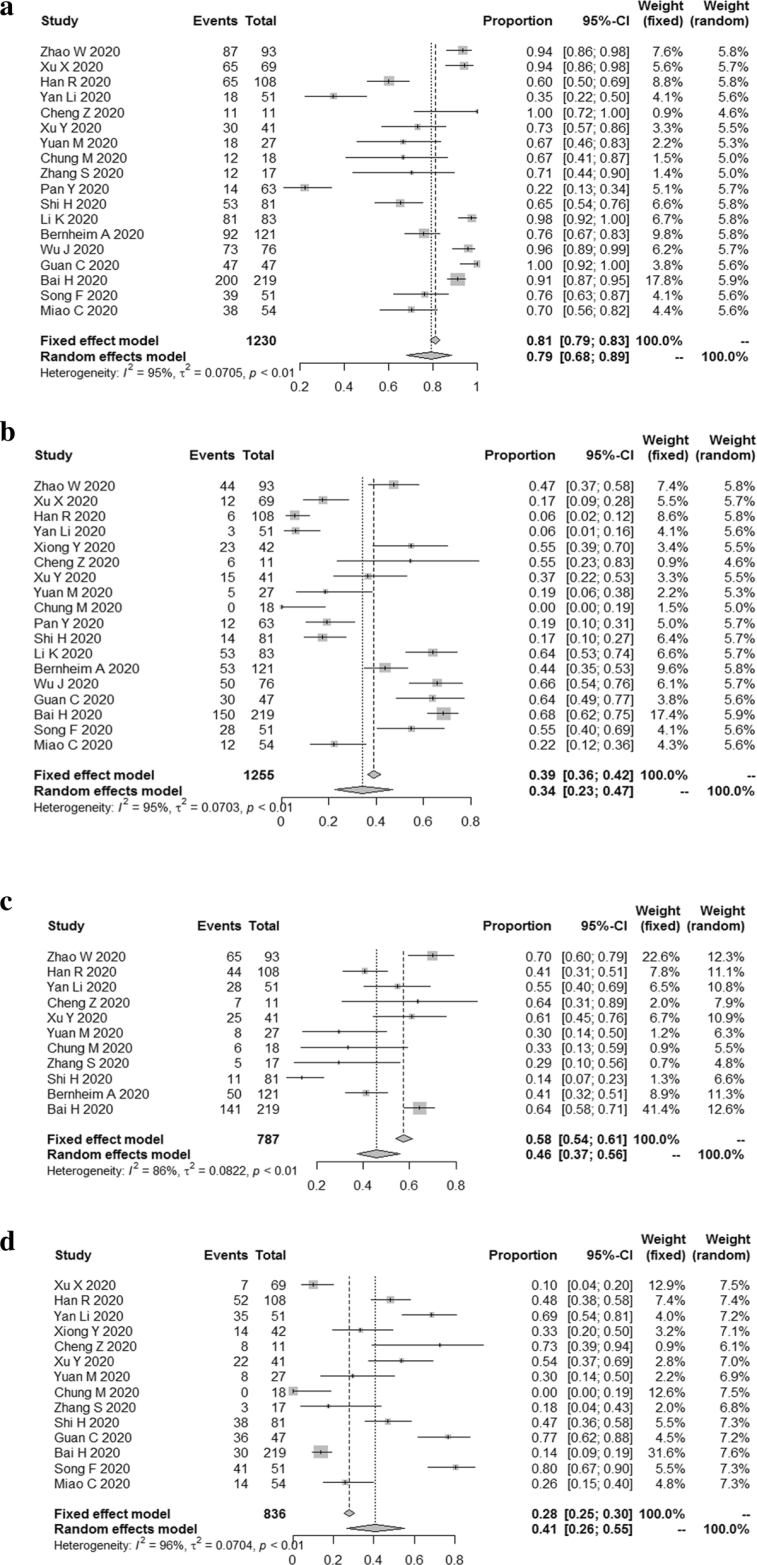
The combined proportion of GGO (**a**), consolidation (**b**), mixed GGO and consolidation (**c**), air bronchogram sign (**d**) in patients with COVID-19

The results showed that the combined proportion of GGO was 0.79 (95% CI 0.68, 0.89) (*I*^*2*^ = 95%, *P* < 0.01) (Fig. 2a), consolidation was 0.34 (95% CI 0.23, 0.47) (*I*^*2*^ = 95%, *P* < 0.01) (Fig. 2b), mixed GGO and consolidation was 0.46 (95% CI 0.37; 0.56) (*I*^*2*^ = 86%, *P* < 0.01) (Fig. 2c), air bronchogram sign was 0.41 (95% CI 0.26; 0.55) (*I*^*2*^ = 96%, *P* < 0.01) (Fig. 2d).

#### The changes of pulmonary interstitial

**Fig. 3:**
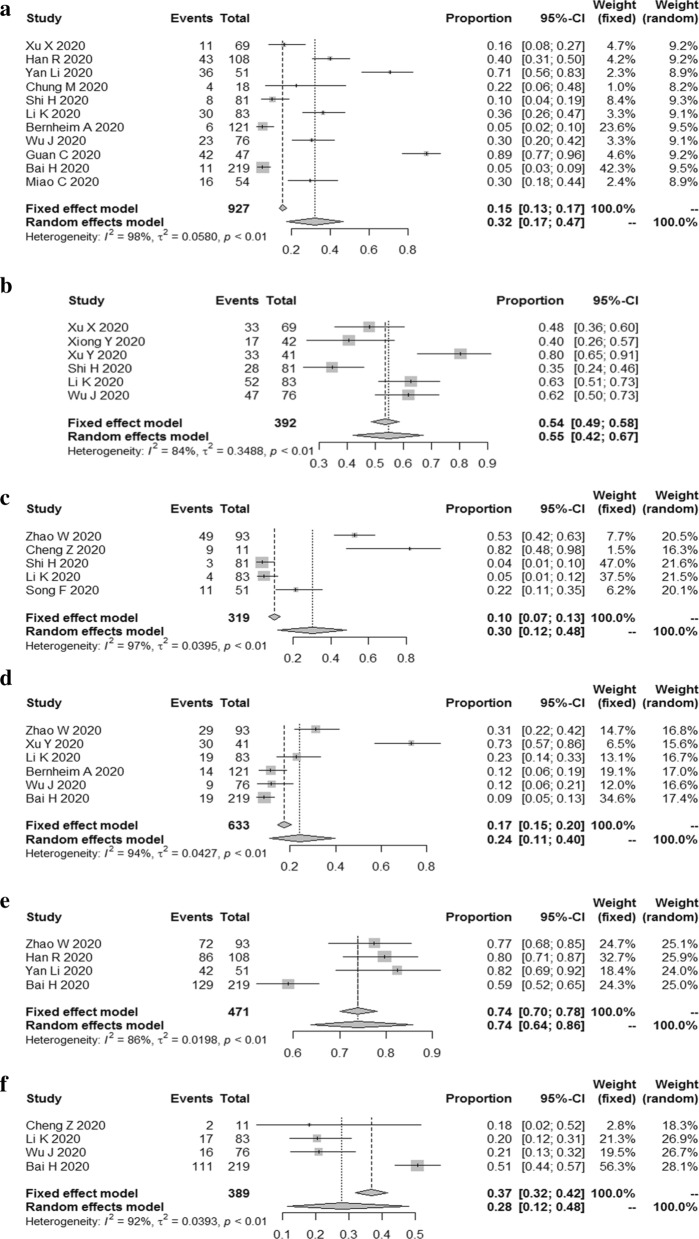
The combined proportion of Crazy paving pattern (**a**), interlobular septal thickening (**b**), reticulation (**c**), bronchial wall thickening (**d**), vascular enlargement (**e**), subpleural linear opacity (**f**) in patients with COVID-19

The results reported that the combined proportion of crazy paving pattern was 0.32 (95% CI 0.17, 0.47) (*I*^*2*^ = 98%, *P* < 0.01) (Fig. 3a); interlobular septal thickening was 0.55 (95% CI 0.42, 0.67) (*I*^*2*^ = 84%, *P* < 0.01) (Fig. 3b); reticulation was 0.30 (95% CI 0.12, 0.48) (*I*^*2*^ = 97%, *P* < 0.01) (Fig. 3c); bronchial wall thickening was 0.24 (95% CI 0.11, 0.40) (*I*^*2*^ = 94%, *P* < 0.01) (Fig. 3d); vascular enlargement was 0.74 (95% CI 0.64, 0.86) (*I*^*2*^ = 86%, *P* < 0.01) (Fig. 3e); subpleural linear opacity was 0.28 (95% CI 0.12, 0.48) (*I*^*2*^ = 92%, *P* < 0.01) (Fig. 3f).

#### Rare signs

**Fig. 4:**
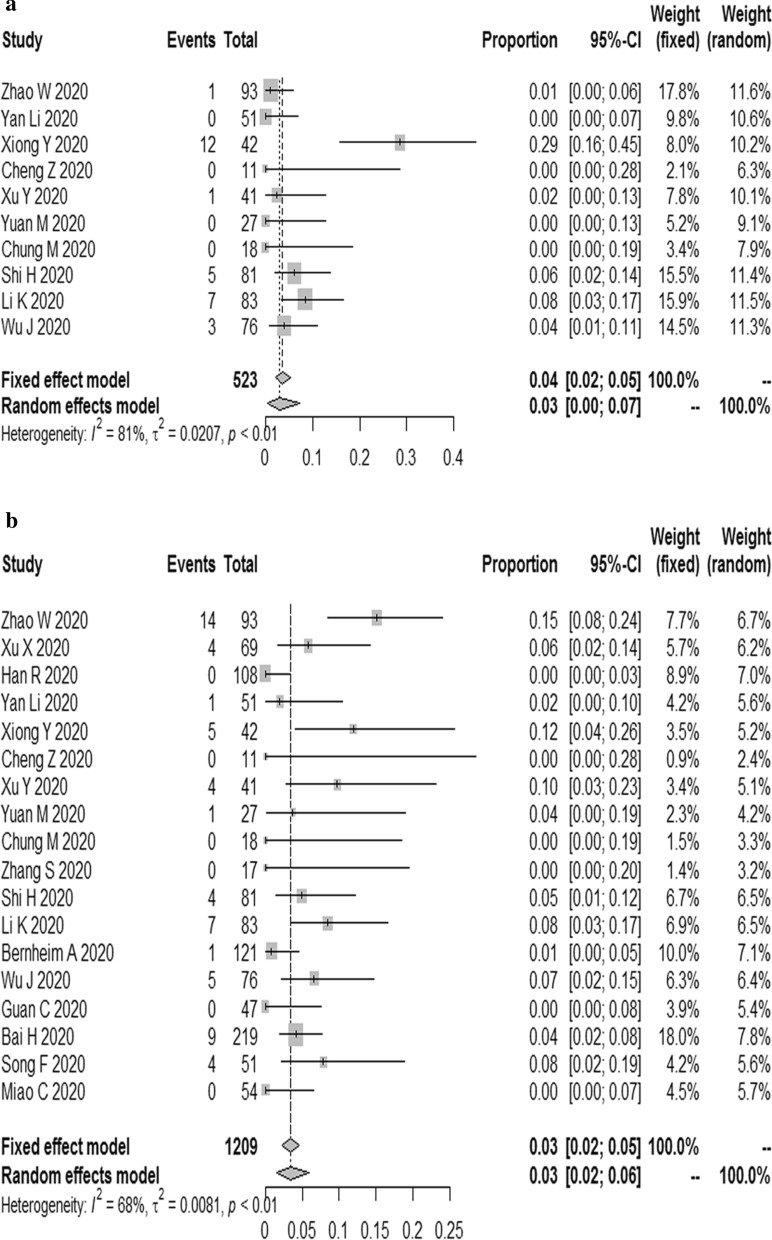
The combined proportion of intrathoracic lymph node enlargment (**a**), pleural effusions (**b**) in patients with COVID-19

The results proved that the combined proportion of intrathoracic lymph node enlargement was 0.03 (95% CI 0.00, 0.07) (*I*^*2*^ = 81%, *P* < 0.01) (Fig. 4a); pleural effusions was 0.03 (95% CI 0.02, 0.06) (*I*^*2*^ = 68%, *P* < 0.01) (Fig. 4b).

#### The lesion distribution in lung

**Fig. 5:**
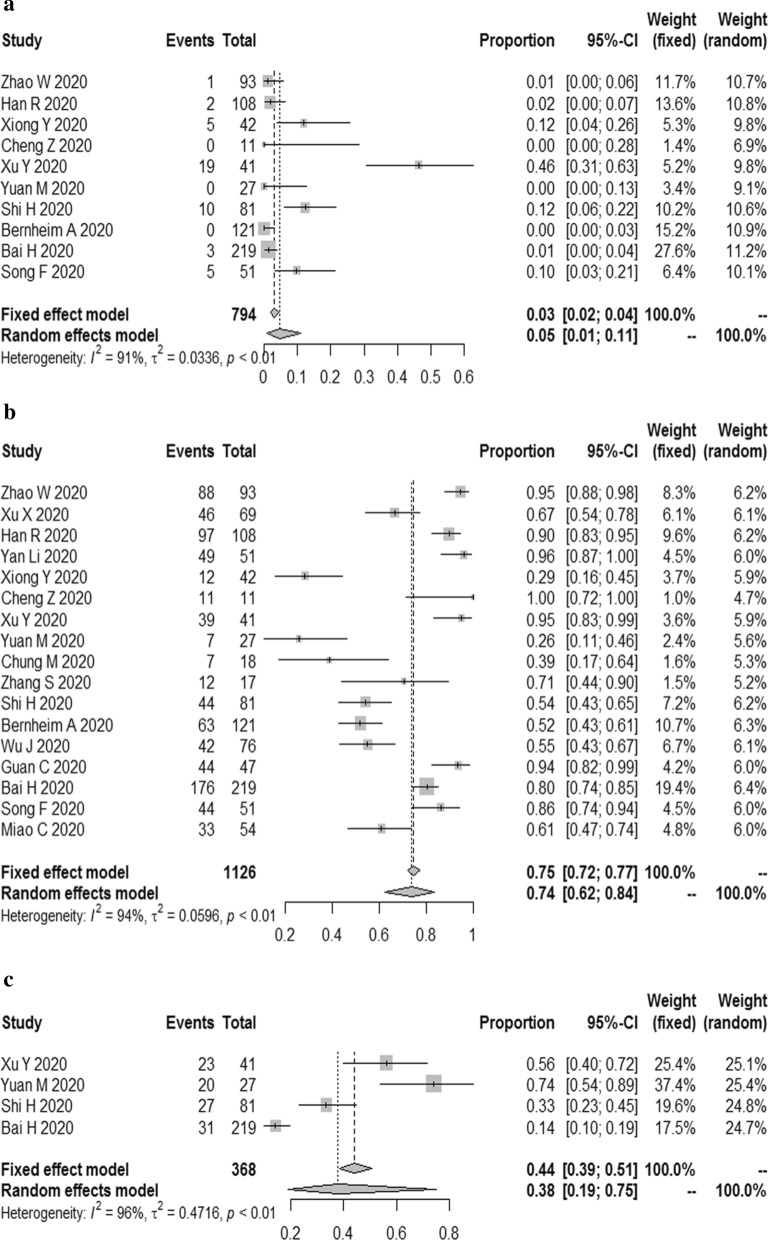
The combined proportion of central (**a**), peripheral (**b**), peripheral involving central (**c**) in patients with COVID-19

**Fig. 6:**
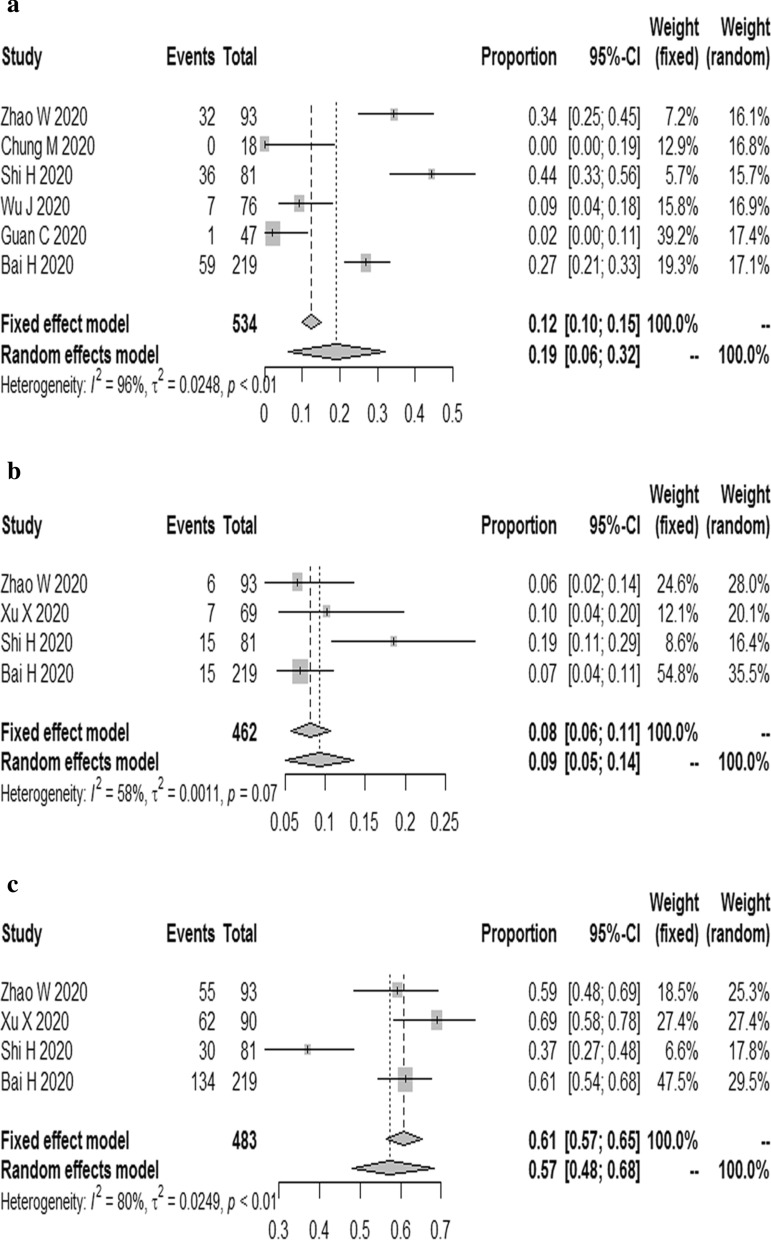
The combined proportion of diffuse (**a**), unifocal involvement (**b**), multifocal involvement (**c**) in patients with COVID-19

**Fig. 7:**
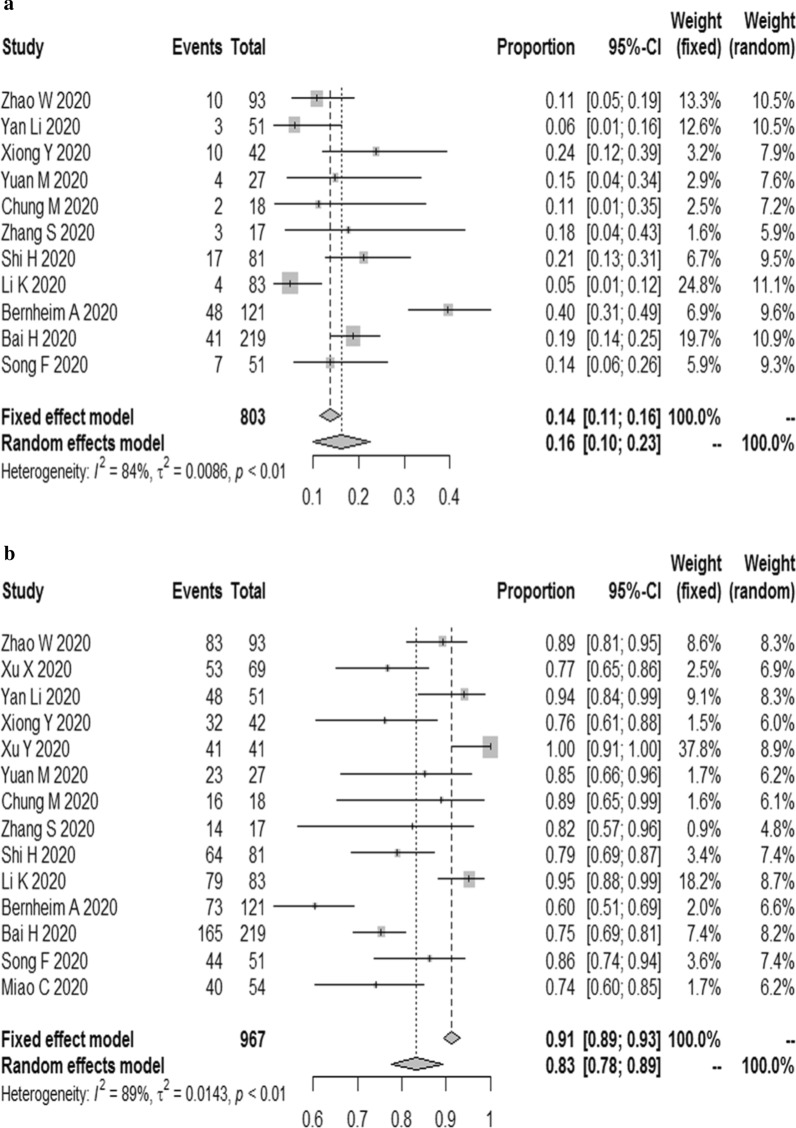
The combined proportion of unilateral (**a**), bilateral (**b**) in patients with COVID-19

**Fig. 8:**
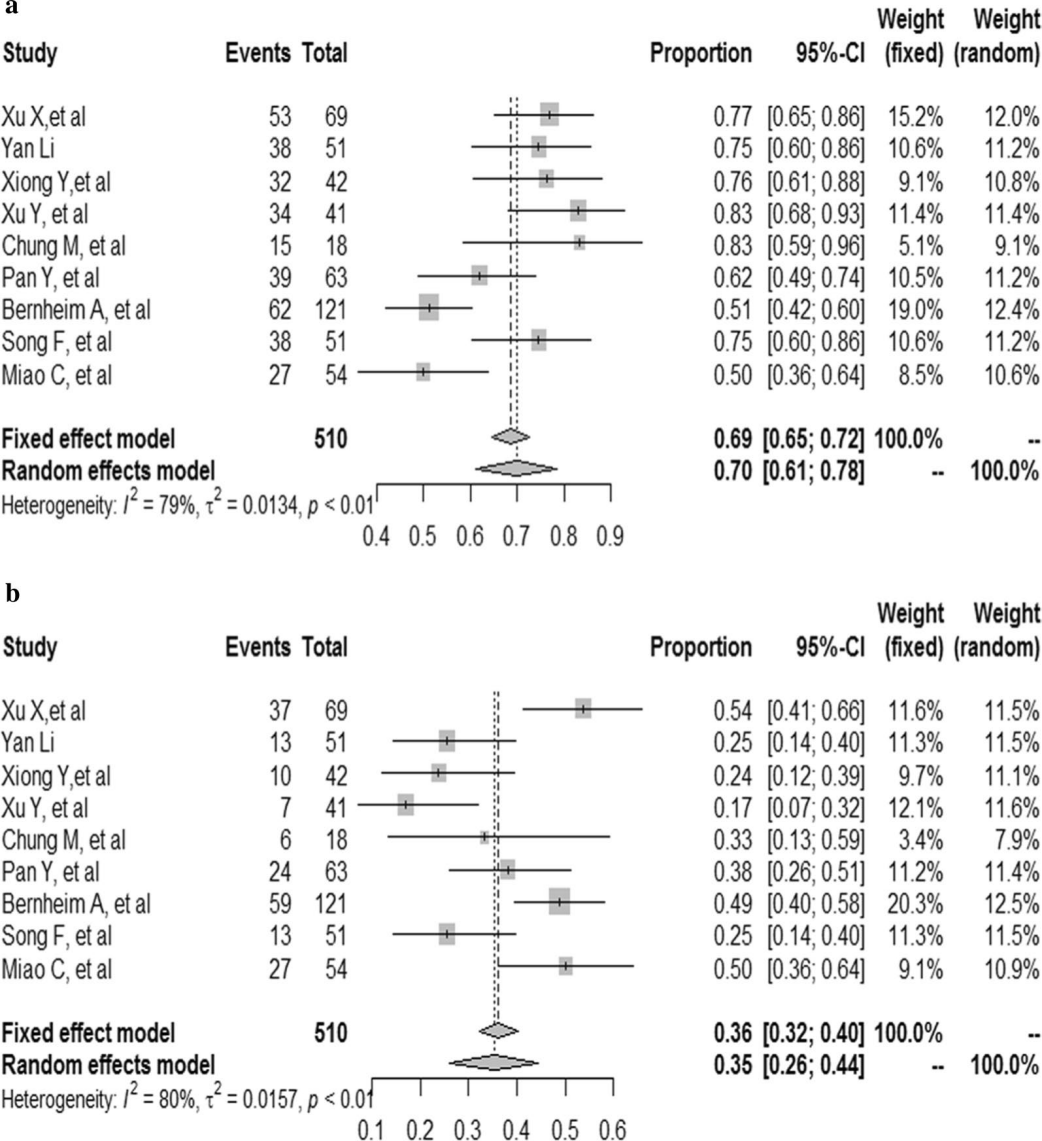
The combined proportion of number of lobes involved (> 2) (**a**), number of lobes involved (≦ 2) (**b**) in patients with COVID-19

The results demonstrated that the combined proportion of central was 0.05 (95% CI 0.01, 0.11) (*I*^*2*^ = 91%, *P* < 0.01) (Fig. 5a); peripheral was 0.74 (95% CI 0.62, 0.84) (*I*^*2*^ = 94%, *P* < 0.01) (Fig. 5b); peripheral involving central was 0.38 (95% CI 0.19, 0.75) (*I*^*2*^ = 96%, *P* < 0.01) (Fig. 5c); diffuse was 0.19 (95% CI 0.06, 0.32) (*I*^*2*^ = 96%, *P* < 0.01) (Fig. 6a); unifocal involvement was 0.09 (95% CI 0.05, 0.14) (*I*^*2*^ = 58%, *P* = 0.07) (Fig. 6b); multifocal involvement was 0.57 (95% CI 0.48, 0.68) (*I*^*2*^ = 80%, *P* < 0.01) (Fig. 6c); unilateral was 0.16 (95% CI 0.10, 0.23) (*I*^*2*^ = 84%, *P* < 0.01) (Fig. 7a); bilateral was 0.83 (95% CI 0.78, 0.89) (*I*^*2*^ = 89%, *P* < 0.01) (Fig. 7b); number of lobes involved (> 2) was 0.70 (95% CI 0.61, 0.78) (*I*^*2*^ = 79%, *P* < 0.01) (Fig. 8a); number of lobes involved (≦ 2) was 0.35 (95% CI 0.26, 0.44) (*I*^*2*^ = 80%, *P* < 0.01) (Fig. 8b).

### Publication bias

A minimum of 10 studies was needed to assess the potential publication bias, therefore we conducted the Egger test on the analyses of GGO, consolidation, mixed GGO and consolidation, crazy paving pattern, air bronchogram sign, pleural effusions, intrathoracic lymph node enlargement, vascular enlargement, peripheral, central, unilateral, bilateral. The results suggested that the publication bias presented in the analyses of mixed GGO and consolidation (*P* = 0.01078), crazy paving pattern (*P* = 0.01784), air bronchogram sign (*P* = 0.01918), bilateral (*P* = 0.001183) (Table 3).

**Table 3:** Egger test for publication bias

CT features	P-value	CT features	*P *value
GGO	0.5425	Intrathoracic lymph node enlargment	0.4386
Consolidation	0.1724	Vascular enlargement	0.1724
Mixed GGO and consolidation	0.01078	Peripheral	0.729
Crazy paving pattern	0.01784	Central	0.3224
Air bronchogram sign	0.01918	Unilateral	0.2431
Pleural effusions	0.9633	Bilateral	0.001183

## Discussion

Our study revealed that GGO, vascular enlargement, interlobular septal thickening more frequently occurred in patients with COVID-19. Peripheral, bilateral, involved lobes > 2 might be the features of SARS-CoV-2 in the distribution aspect. In this study, intrathoracic lymph node enlargement, pleural effusions, the lesion distribution in lung of central, unifocal, and unilateral were not frequently observed. Therefore, based on the above features of COVID-19 in chest CT imaging, it might be a promising means for identifying COVID-19.

This novel coronavirus disease is known as COVID-19 by the world health organization [30, 31]. Early detection, early diagnosis, early isolation and early treatment principle are important to control this disease. RT-PCR is the reference standard [6, 7]. But it requires a laboratory-level facility, reliable power supply, expensive equipment and trained personnel to properly conduct RT-PCR tests, which limits its application to some extent [32]. In addition to the nucleic acid test, CT also can be helpful to diagnose COVID-19. The diagnosis of viral pneumonia based on radiologic features by radiologists as one of the diagnostic criteria for COVID-19 according to the diagnosis and treatment program (6th version) published by the National Health Commission of the People’s Republic of China [14, 33]. High-resolution CT is highly sensitive to detect lung abnormalities, which is quite helpful for early diagnosis of the disease that can trigger early treatment and facilitates to contain this emergency disease [19, 34, 35]. Some articles have shown the detailed CT features of COVID-19 [6, 19, 36]. Our study may conclude some common CT imaging features in patients affected by SARS-CoV-2 pneumonia.

The CT patterns of viral pneumonia are related to the pathogenesis of viral infection. Viruses from the same family (e.g. Coronaviridae) have similar pathogenesis [37]. The SARS-CoV-2 belongs to the family Coronaviridae, which includes other viruses like SARS-associated coronavirus (SARS-CoV) and Middle East respiratory syndrome coronavirus (MERS-CoV) [30]. Some investigations [8, 28, 36] have shown that COVID-19 pneumonia CT findings were partially similar to other viral pneumonia, like SARS, MERS and H7N9 pneumonia [38–40]. The pathological changes included thickening of the basement membrane of the alveoli capillary, edema of the alveoli septum, pulmonary hyaline membrane formation, inflammatory cell infiltration and inflammatory edema, pulmonary interstitial hyperplasia and fibrosis, apoptosis of alveolar epithelium cells [41, 42]. Based on the image characteristic of SARS-CoV-2 pneumonia which had been reported in some articles [41–43], the pathology of GGOs may be the thickening of alveolar wall, collapse of alveolar cavity, reduction of air content in alveolar cavity and inflammatory cells infiltration or a combination of these features. We estimated the pathological mechanism of COVID-19 includes bronchoalveolar destruction and damage to lung parenchyma near the bronchioles in the early stages [43]. In the late stage, diffuse alveolar injury and acute fibrous and organic pneumonia can be observed [44]. This pathological pattern is the same as imaging pattern like GGO at first and then consolidation dense consolidative lesions, early in the disease. With the progression of the disease, lesions often turn more linear fashion with a predilection for the lung periphery (and somewhat with a “crazy” paving pattern or emergence of a “reverse halo” sign).

We also observed some interstitial changes in patients with COVID-19. Hitherto, some autopsy cases have revealed the pathological features of COVID-19 [43, 45–47]. The pathological changes of pulmonary fibrosis injury in COVID-19 include extensive diffuse alveolar injury with bilateral edema, protein or fibrin exudation, and diffuse reactive proliferation of type II pneumocytes. It was even can be observed that interstitial fibroblast proliferation caused alveolar septa to thicken, forming hyaline membranes consistent with fibrosis. These pathological changes may be due to a disruption of the ACE/ACE2 (angiotensin-converting enzyme/ angiotensin-converting enzyme 2) balance [48–53], which were presented as the crazy paving-pattern, interlobular septal thickening, bronchial wall thickening on chest CT.

Lymphadenopathy and pleural effusions are atypical imaging features in COVID-19 patients. Severe/critical patients showed more lymph node enlargement, and pleural effusion [8, 22]. Li et al. [29] reported that lymphadenopathy and pleural effusions were poor prognostic indicators according to his logistic regression models in COVID-19 pneumonia. Lymphadenopathy may be related to the immune response. The immune response of patients with COVID-19 pneumonia is stronger than that of other viral pneumonia, especially those with moderate to severe COVID-19 pneumonia [29]. Similarly, lymphadenopathy and pleural effusions are important predictors of an unfavorable outcome in patients infected with MERS­CoV or avian influenza H5N1 [54–56]. However, articles on the above two imaging changes are less, and more evidence is needed to verify these conclusions.

Pulmonary lesions were most commonly in the peripheral, which was related to ACE2 [57]. ACE2 has been established as a functional receptor for SARS-CoV, which plays a crucial role in the pathogenesis of COVID-19 [58]. ACE2 was abundantly expressed on the surface of alveolar type II pneumocytes and the capillary endothelial cells [57, 59], where are the targets of viral entry and replication. As the virus invades, type II pneumocytes and capillary endothelial cells are constantly destroyed, which may explain why most lesions are located peripherally.

According to the results of most articles, lesions are mostly distributed peripherally in the lung, which facilitates detection by lung ultrasonography (US) [8, 60]. Xing et al. found abnormal US results in patients with COVID-19 pneumonia: mainly B-lines [61]. In that study, US examinations was performed at different stages of disease, B-lines showed parallel changes with the clinical severity. However, the unspecific abnormalities realizations in chest CT increase with age, and similarly, the number of chest areas positive for B-lines increases in the elderly [62]. Age-related confounding factors should be taken into consideration in clinical practice in order to avoid misdiagnoses.

Most of the literature has focused on CT manifestations of COVID-19, however, due to limited CT availability in regions of the world, infection control issues related to access to CT rooms, the inefficiencies in decontamination, and the reduction in radiological service availability due to the widespread use of CT for the diagnosis and follow up of COVID-19, there is the need for alternative diagnostic tools. The US could be proposed for the diagnosis or follow up to minimize the risk of cross-infection. Additionally, the US may also play a vital role in areas around the world with limited access to other diagnostic tools, even in austere environments [62].

Nevertheless, we also encountered some limitations: (1) eligible studies were retrospective studies; (2) large heterogeneity among included articles might affect the reliability and stability of results we analyzed to some extent.

## Conclusions

GGO, vascular enlargement, interlobular septal thickening more frequently occurred in patients with COVID-19. Peripheral, bilateral, involved lobes > 2 might be the features of COVID-19 in the distribution aspect. Based on the above features of COVID-19 in chest CT imaging, it might be a promising means for identifying COVID-19.

## Supplementary information

**Additional file 1.** The exact search string and settings for each database.

**Additional file 2.** The 36 references of exclusion.

## Data Availability

Not applicable.

## Abbreviations

COVID-19: Coronavirus disease 2019
PHEIC: Public health emergency of international concern
CT: Computer tomography
GGO: Ground-glass opacities
SARS: Severe acute respiratory syndrome
MERS: Middle East respiratory syndrome
ACE: Angiotensin-converting enzyme
ACE2: Angiotensin-converting enzyme 2
