# Supplementary material for: The chest CT features of coronavirus disease 2019 (COVID-19) in China: a meta-analysis of 19 retrospective studies
Source: Virol J. 2020 Oct 21;17:159. doi: 10.1186/s12985-020-01432-9 (PMC7576554; doi:10.1186/s12985-020-01432-9)
Supplement: Supplementary file 1 — Additional file 1. The exact search string and settings for each database. [file 12985_2020_1432_MOESM1_ESM.docx]

**Supplementary materia 1**. The exact search string and settings for each database.

**PubMed**

#1 **COVID-19,:** (((((("covid 19"[All Fields] OR "covid 2019"[All Fields]) OR "severe acute respiratory syndrome coronavirus 2"[Supplementary Concept]) OR "severe acute respiratory syndrome coronavirus 2"[All Fields]) OR "2019 ncov"[All Fields]) OR "sars cov 2"[All Fields]) OR "2019ncov"[All Fields]) OR ((("coronavirus"[MeSH Terms] OR "coronavirus"[All Fields]))

#2 **SARS-COV-2,:**"severe acute respiratory syndrome coronavirus 2"[Supplementary Concept] OR "severe acute respiratory syndrome coronavirus 2"[All Fields] OR "sars cov 2"[All Fields]

**#**3 **search (#1 OR #2)**

#4 **COVID-19,CT,:**((((((("covid 19"[All Fields] OR "covid 2019"[All Fields]) OR "severe acute respiratory syndrome coronavirus 2"[Supplementary Concept]) OR "severe acute respiratory syndrome coronavirus 2"[All Fields]) OR "2019 ncov"[All Fields]) OR "sars cov 2"[All Fields]) OR "2019ncov"[All Fields]) OR ((("coronavirus"[MeSH Terms] OR "coronavirus"[All Fields])) AND ((("j comput tomogr"[Journal] OR "commun theory"[Journal]) OR "cancer ther"[Journal]) OR "ct"[All Fields])

#5 **SARS-COV-2,CT,:**(("severe acute respiratory syndrome coronavirus 2"[Supplementary Concept] OR "severe acute respiratory syndrome coronavirus 2"[All Fields]) OR "sars cov 2"[All Fields]) AND ((("j comput tomogr"[Journal] OR "commun theory"[Journal]) OR "cancer ther"[Journal]) OR "ct"[All Fields])

#6 **search (#4 OR #5)**

#7 **COVID-19,China,:**((((((("covid 19"[All Fields] OR "covid 2019"[All Fields]) OR "severe acute respiratory syndrome coronavirus 2"[Supplementary Concept]) OR "severe acute respiratory syndrome coronavirus 2"[All Fields]) OR "2019 ncov"[All Fields]) OR "sars cov 2"[All Fields]) OR "2019ncov"[All Fields]) OR ((("coronavirus"[MeSH Terms] OR "coronavirus"[All Fields])) AND ((("j comput tomogr"[Journal] OR "commun theory"[Journal]) OR "cancer ther"[Journal]) OR "ct"[All Fields]) AND ((("china"[MeSH Terms] OR "china"[All Fields]) OR "china s"[All Fields]) OR "chinas"[All Fields])

#8 **SARS-COV-2,China,:**(("severe acute respiratory syndrome coronavirus 2"[Supplementary Concept] OR "severe acute respiratory syndrome coronavirus 2"[All Fields]) OR "sars cov 2"[All Fields]) AND ((("j comput tomogr"[Journal] OR "commun theory"[Journal]) OR "cancer ther"[Journal]) OR "ct"[All Fields]) AND ((("china"[MeSH Terms] OR "china"[All Fields]) OR "china s"[All Fields]) OR "chinas"[All Fields])

#9 **search (#7 OR #8)**

#10 **search (#3 AND #6 AND** #9**)**

**EMBASE**

#1 **COVID-19**:'covid 19'/exp OR 'covid 19'

#2 **SARS-COV-2**:'sars cov 2'/exp OR 'sars cov 2'

#3 **COVID-19 CT**:'covid-19 ct' OR (('covid 19'/exp OR 'covid 19') AND ('ct'/exp OR ct))

#4 **SARS-COV-2 CT**: 'sars-cov-2 ct' OR (('sars cov 2'/exp OR 'sars cov 2') AND ('ct'/exp OR ct))

#5 **COVID-19 China**:'covid-19 china' OR (('covid 19'/exp OR 'covid 19') AND ('china'/exp OR china))

#6 **SARS-COV-2 China**:'sars-cov-2 china' OR (('sars cov 2'/exp OR 'sars cov 2') AND ('china'/exp OR china))

#7 **SARS-COV-2 Chinese**:'sars-cov-2 chinese' OR (('sars cov 2'/exp OR 'sars cov 2') AND ('chinese'/exp OR chinese))

#8 **COVID-19 Chinese:**'covid-19 chinese' OR (('covid 19'/exp OR 'covid 19') AND ('chinese'/exp OR chinese))

#9 **COVID-19 CT** **China**:'covid-19 ct china' OR (('covid 19'/exp OR 'covid 19') AND ('ct'/exp OR ct) AND ('china'/exp OR china))

#10 **SARS-COV-2 CT China**: 'sars-cov-2 ct china' OR (('sars cov 2'/exp OR 'sars cov 2') AND ('ct'/exp OR ct) AND ('china'/exp OR china))

**Web of science**

#1 **COVID-19**:TS=( COVID-19)

#2 **SARS-COV-2**:TS=(SARS-COV-2)

#3 **COVID-19 CT**:TS=(COVID-19 CT)

#4 **SARS-COV-2 CT**: TS=(SARS-COV-2 CT)

#5 **COVID-19 China:**TS=(COVID-19 China)

#6 **SARS-COV-2 China**:TS=(SARS-COV-2 China)

#7 **SARS-COV-2 Chinese:**TS=(SARS-COV-2 Chinese)

#8 **COVID-19 Chinese**: TS=(COVID-19 Chinese)

#9 **COVID-19 CT China**:TS=(COVID-19 CT China)

#10 **SARS-COV-2 CT China**: TS=(SARS-COV-2 CT China)
