# Supplementary material for: The chest CT features of coronavirus disease 2019 (COVID-19) in China: a meta-analysis of 19 retrospective studies
Source: Virol J. 2020 Oct 21;17:159. doi: 10.1186/s12985-020-01432-9 (PMC7576554; doi:10.1186/s12985-020-01432-9)
Supplement: Supplementary file 2 — Additional file 2. The 36 references of exclusion. [file 12985_2020_1432_MOESM2_ESM.docx]

**Supplementary materia 2**. The 36 references of exclusion.

1. Lin C, Ding Y, Xie B, Sun Z, Li X, Chen Z, et al. Asymptomatic novel coronavirus pneumonia patient outside Wuhan: The value of CT images in the course of the disease. Clin Imaging. 2020;63:7-9.

2. Lu T, Pu H. Computed Tomography Manifestations of 5 Cases of the Novel Coronavirus Disease 2019 (COVID-19) Pneumonia From Patients Outside Wuhan. J Thorac Imaging. 2020;35(3):W90-3.

3. Shi H, Han X, Zheng C. Evolution of CT Manifestations in a Patient Recovered from 2019 Novel Coronavirus (2019-nCoV) Pneumonia in Wuhan, China. Radiology. 2020;295(1):20.

4. Zhang W. Imaging changes in severe COVID-19 pneumonia. Intensive Care Med.2020;46(4):583-5.

5. Duan YN, Qin J. Pre- and Posttreatment Chest CT Findings: 2019 Novel Coronavirus (2019-nCoV) Pneumonia.Radiology.2020;295(1):21.

6. Shi F, Yu Q, Huang W, Tan C. 2019 Novel Coronavirus (COVID-19) Pneumonia with Hemoptysis as the Initial Symptom: CT and Clinical Features. Korean J Radiol. 2020;21(5):537-40.

7. Qu J, Yang R, Song L, Kamel IR. Atypical lung feature on chest CT in a lung adenocarcinoma cancer patient infected with COVID-19. Ann Oncol. 2020.31(6):825-6.

8. Sun Q, Xu X, Xie J, Li J, Huang X. Evolution of Computed Tomography Manifestations in Five Patients Who Recovered from Coronavirus Disease 2019 (COVID-19) Pneumonia. Korean J Radiol. 2020;21(5):614-9.

9. Lei P, Fan B, Yuan Y. The evolution of CT characteristics in the patients with COVID-19 pneumonia. J infection. 2020;80(6):e29.

10. Lei P, Fan B, Mao J, Wei J, Wang P. The progression of computed tomographic (CT) images in patients with coronavirus disease (COVID-19) pneumonia: Running title: The CT progression of COVID-19 pneumonia. J Infect. 2020;80(6):e30-1.

11. Lei J, Li J, Li X, Qi X. CT Imaging of the 2019 Novel Coronavirus (2019-nCoV) Pneumonia. Radiology. 2020;295(1):18.

12. Wei J, Xu H, Xiong J, Shen Q, Fan B, Ye C, et al. 2019 Novel Coronavirus (COVID-19) Pneumonia: Serial Computed Tomography Findings. Korean J Radiol. 2020;21(4):501-4.

13. Chen R, Chen J, Meng QT. Chest computed tomography images of early coronavirus disease (COVID-19). Can J Anaesth. 2020;67(6):754-5.

14. Liu H, Liu F, Li J, Zhang T, Wang D, Lan W. Clinical and CT imaging features of the COVID-19 pneumonia: Focus on pregnant women and children. J Infect. 2020;80(5):e7-13.

15. Xia W, Shao J, Guo Y, Peng X, Li Z, Hu D. Clinical and CT features in pediatric patients with COVID-19 infection: Different points from adults. Pediatr Pulmonol.2020;55(5):1169-74.

16. Li W, Cui H, Li K, Fang Y, Li S. Chest computed tomography in children with COVID-19 respiratory infection. Pediatr Radiol. 2020;50(6):796-9.

17. Liu D, Li L, Wu X, Zheng D, Wang J, Yang L, et al. Pregnancy and Perinatal Outcomes of Women With Coronavirus Disease (COVID-19) Pneumonia: A Preliminary Analysis. AJR Am J Roentgenol.2020;215(1):127-32.

18. Yang W, Cao Q, Qin L, Wang X, Cheng Z, Pan A, et al. Clinical characteristics and imaging manifestations of the 2019 novel coronavirus disease (COVID-19):A multi-center study in Wenzhou city, Zhejiang, China. J Infect.2020;80(4):388-93.

19. Li K, Fang Y, Li W, Pan C, Qin P, Zhong Y, et al. CT image visual quantitative evaluation and clinical classification of coronavirus disease (COVID-19). Eur Radiol. 2020;30(8):4407-16.

20. Ai T, Yang Z, Hou H, Zhan C, Chen C, Lv W, et al. Correlation of Chest CT and RT-PCR Testing for Coronavirus Disease 2019 (COVID-19) in China: A Report of 1014 Cases. Radiology. 2020;296(2):E32-40.

21. Li L, Qin L, Xu Z, Yin Y, Wang X, Kong B, et al. Using Artificial Intelligence to Detect COVID-19 and Community-acquired Pneumonia Based on Pulmonary CT: Evaluation of the Diagnostic Accuracy. Radiology.2020;296(2):E65-71.

22. Luo L, Luo Z, Jia Y, Zhou C, He J, Lyu J, et al. CT differential diagnosis of COVID-19 and non-COVID-19 in symptomatic suspects: a practical scoring method. BMC Pulm Med.2020;20(1):129.

23. Chen H, Ai L, Lu H, Li H. Clinical and imaging features of COVID-19. Radiol Infect Dis. 2020;7(2):43-50.

24. Yang S, Shi Y, Lu H, Xu J, Li F, Qian Z, et al. Clinical and CT features of early stage patients with COVID-19: a retrospective analysis of imported cases in Shanghai, China. Eur Respir J. 2020;55(4).

25. Huang Z, Zhao S, Xu L, Chen J, Lin W, Zeng H, et al. Imaging features and mechanisms of novel coronavirus pneumonia (COVID-19): Study Protocol Clinical Trial (SPIRIT Compliant). Medicine (Baltimore).2020;99(16):e19900.

26. Waller JV, Kaur P, Tucker A, Lin KK, Diaz MJ, Henry TS, et al. Diagnostic Tools for Coronavirus Disease (COVID-19): Comparing CT and RT-PCR Viral Nucleic Acid Testing. AJR Am J Roentgenol. 2020:1-5.

27. Wang L, Gao YH, Lou LL, Zhang GJ. The clinical dynamics of 18 cases of COVID-19 outside of Wuhan, China. Eur Respir J. 2020;55(4).2000398.

28. Huang C, Wang Y, Li X, Ren L, Zhao J, Hu Y, et al. Clinical features of patients infected with 2019 novel coronavirus in Wuhan, China. Lancet.2020;395(10223):497-506.

29. Sun D, Li H, Lu XX, Xiao H, Ren J, Zhang FR, et al. Clinical features of severe pediatric patients with coronavirus disease 2019 in Wuhan: a single center's observational study. World J Pediatr.2020;16(3):251-9.

30. Xu X, Yu C, Qu J, Zhang L, Jiang S, Huang D, et al. Imaging and clinical features of patients with 2019 novel coronavirus SARS-CoV-2. Eur J Nucl Med Mol Imaging. 2020;47(5):1275-80.

31. Tian S, Hu N, Lou J, Chen K, Kang X, Xiang Z, et al. Characteristics of COVID-19 infection in Beijing. J Infection. 2020;80(4):401-6.

32. Chen N, Zhou M, Dong X, Qu J, Gong F, Han Y, et al. Epidemiological and clinical characteristics of 99 cases of 2019 novel coronavirus pneumonia in Wuhan, China: a descriptive study. Lancet. 2020;395(10223):507-13.

33. Qian GQ, Yang NB, Ding F, Ma AHY, Wang ZY, Shen YF, et al. Epidemiologic and clinical characteristics of 91 hospitalized patients with COVID-19 in Zhejiang, China: a retrospective, multi-centre case series. QJM. 2020;113(7):474-81.

34. Colombi D, Bodini FC, Petrini M, Maffi G, Morelli N, Milanese G, et al. Well-aerated Lung on Admitting Chest CT to Predict Adverse Outcome in COVID-19 Pneumonia. Radiology. 2020;296(2):E86-96.

35. Bernheim A, Mei X, Huang M, Yang Y, Fayad ZA, Zhang N, et al. Chest CT Findings in Coronavirus Disease-19 (COVID-19): Relationship to Duration of Infection. Radiology. 2020;295(3):200463.

36. Burhan E, Prasenohadi P, Rogayah R, Isbaniyah F, Reisa T, Dharmawan I. Clinical Progression of COVID-19 Patient with Extended Incubation Period, Delayed RT-PCR Time-to-positivity, and Potential Role of Chest CT-scan. Acta Med Indones. 2020;52(1):80-3.
